# Questionable Advertising of Psychotropic Medications and Disease Mongering

**DOI:** 10.1371/journal.pmed.0030321

**Published:** 2006-07-25

**Authors:** Jeffrey R Lacasse, Jonathan Leo

**Affiliations:** **1**Florida State UniversityTallahassee, FloridaUnited States of America; **2**Lincoln Memorial UniversityHarrogate, TennesseeeUnited States of America

David Healy raises intriguing questions regarding the rapid increase in bipolar diagnoses and the use of “mood stabilizing” medications [ 1]. Although this phenomenon is multifactorial, surely consumer advertising has played a role.

A widely disseminated advertising campaign for aripiprazole (Abilify) claimed that it worked in the brain “like a thermostat to restore balance” [ 2]. Interestingly, the Abilify product Web sites for schizophrenia and bipolar disorder both used virtually identical explanations to describe both neuropathology and the drug's mechanism of action. Print advertisements promoting aripiprazole for bipolar disorder claimed: “When activity of key brain chemicals is too high, Abilify lowers it…. When activity of key brain chemicals is too low, Abilify raises it” [ 3].

Since the product information insert approved by the United States Food and Drug Administration (FDA) lists the mechanism of action as “unknown” [ 4], this advertisement is debatable. It is further questionable whether the complexities of treating bipolar disorder (with its unknown etiology and well-known heterogeneity in response to treatment) are accurately portrayed as a reliable, mechanical thermostat. However, consumers are likely to find such advertisements compelling.

Regarding unipolar depression, we recently argued [ 5] that antidepressant manufacturers commonly advertise their products by claiming that depression is caused by a lack of serotonin and that selective serotonin reuptake inhibitors normalize this deficiency, a claim not congruent with the peer-reviewed literature or FDA-approved product information. We have not received any academic objections to our article, but several prominent psychiatrists have affirmed our conclusions. For instance, Wayne Goodman, Chair of the FDA Psychopharmacological Advisory Committee, admitted that the serotonergic theory of depression is a “useful metaphor”—and one that he never uses within his own psychiatric practice [ 6].

The presentation of metaphorical explanations as scientific consensus in consumer advertising has not been publicly addressed by the relevant professional associations. In fact, we observe that a cooperative relationship exists between industry and medical facilities, even highly esteemed ones: the Mayo Clinic Web site on depression, sponsored by Wyeth Pharmaceuticals (makers of venlafaxine) explains the treatment of depression via the serotonin metaphor [ 7].

Such bioreductionistic and highly arguable advertisements for psychiatric treatments imply much about the disorder they are licensed for. As Dr. Healy suggests, consumers who view such advertisements are likely to characterize their problems in a manner congruent with industry promotion and to request well-advertised pharmaceuticals as treatment. At a bare minimum, increased medicalization will result; in some cases, disease mongering may indeed be an appropriate characterization.

Such consumer advertising is only possible in the absence of vigorous government regulation [ 8] or outcry from professional associations. We hypothesize that their combined silence significantly contributes to the process of disease mongering.
